# Neonatal Sacrococcygeal Neuroblastoma Mimicking a Teratoma

**DOI:** 10.1155/2017/3624847

**Published:** 2017-01-01

**Authors:** Leticia Gely, Humberto Lugo-Vicente, María Correa-Rivas, Kary Bouet, Zayhara Reyes Bou, Mohammed Suleiman, Inés García

**Affiliations:** ^1^Department of Neonatal-Perinatal Medicine, University of Puerto Rico School of Medicine, San Juan, PR, USA; ^2^Department of Pediatric Surgery, University of Puerto Rico School of Medicine, San Juan, PR, USA; ^3^Department of Pathology, University of Puerto Rico School of Medicine, San Juan, PR, USA

## Abstract

We reported the first case of a congenital intrapelvic presacral neuroblastoma in Puerto Rico managed in the early neonatal period. The preoperative diagnosis was a sacrococcygeal teratoma Altman stage IV classification. This case confirms the importance of a comprehensive physical examination and observation of low-risk newborn infants with a history of adequate prenatal care and an unremarkable fetal ultrasonogram during pregnancy.

## 1. Introduction

Neonatal tumors are rare [1]. Around 2% of all pediatric malignancies are neonatal tumors, with an incidence of 1.58 to 3.65 per 100,000 live births [2]. The most common neonatal tumor is neuroblastoma, accounting for 28–39% of tumors in this period, with an estimated incidence of 0.61 per 100,000 live births [3]. They originate from the neural crest cells which normally give rise to the adrenal medulla and sympathetic ganglia [4].

Clinical presentation of neuroblastoma is dependent upon the site of the tumor origin, disease extent, and the presence of paraneoplastic syndromes [5]. The majority of tumors (65%) arise in the abdomen, with over half of these arising in the adrenal gland [5]. Additional sites of origin include the neck, chest, and pelvis. Metastatic lesions are common presenting findings of neuroblastoma, especially in the neonate [5]. Disease dissemination occurs through lymphatic and hematogenous routes. Bone, bone marrow, and liver are the most common sites of hematogenous spread with particular predilection for metaphyseal, skull, and orbital bone sites [5]. The prognosis for children with neuroblastoma is inversely correlated to the age of the child at diagnoses and the extent of the disease [4].

Mothers who delivered infants diagnosed with neuroblastoma can develop symptoms during their third trimester such as sweating, pallor, headaches, palpitations, tingling of hands and feet, and/or hypertension. This occurs by transplacental exchange of fetal tumor catecholamines into the maternal circulation [4].

We presented a newborn with an intrapelvic neuroblastoma originally thought to be an Altman stage IV intrapelvic sacrococcygeal teratoma.

## 2. Case Presentation

This is a case of a term male born at 39 1/7 weeks of gestational age by spontaneous vaginal delivery to an 18-years-old mother G_2_P_1_A_0_. The mother had no history of systemic illness. There was no consanguinity in the family. Prenatal screening tests (Human Immunodeficiency Virus, Hepatitis B, and Syphilis) were negative; Group B Streptococcus was positive; the mother did not receive antenatal antibiotics during pregnancy. No prenatal complications were reported, and no fetal anomalies or abdominal findings were detected on antenatal ultrasound. Patient had an Apgar 8 at one minute and 9 at 5 minutes. No complications during labor and delivery were reported.

**Figure 1:**
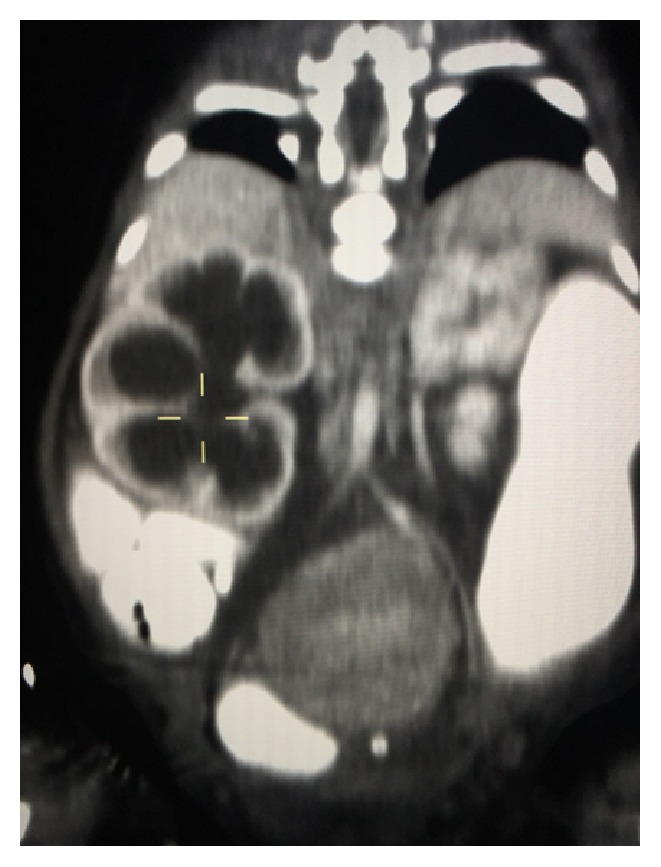
CT image showing mass on presacral space and hydroureteronephrosis.

**Figure 2:**
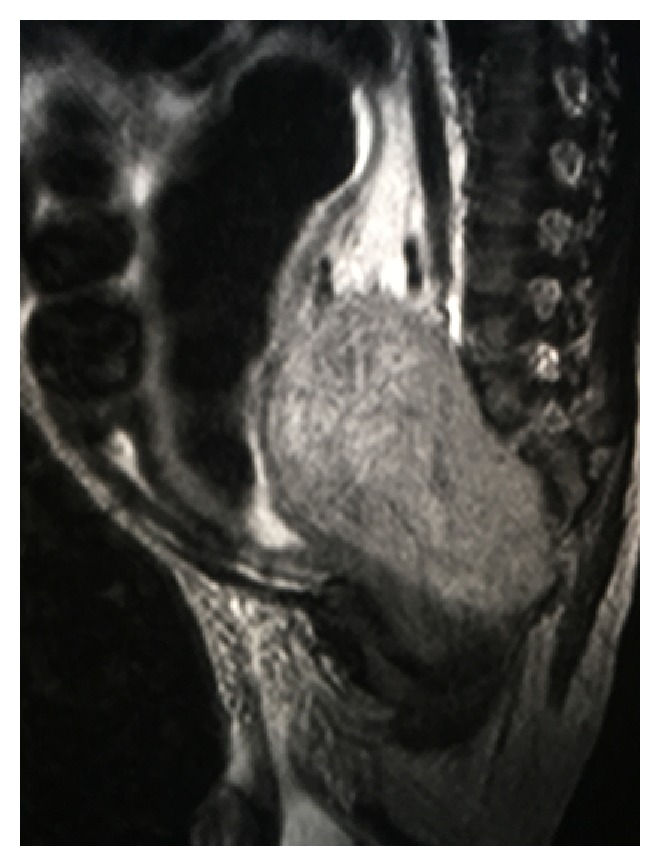
MRI image showing mass extending to sacral vertebras.

At first day of life, the patient presented with hypoactivity, poor sucking, and cyanosis for which he was admitted to the Neonatal Intensive Care Unit due to suspected early onset of neonatal sepsis. Diagnostic studies were performed and therapeutic management was instituted, including antibiotic coverage. On day #2 of life, the patient presented with marked abdominal distention and marked bright red bloody stools. Oral feedings were discontinued, an orogastric tube was placed for gastrointestinal decompression, and metronidazole therapy was added to antibiotic therapy. The abdominal radiography revealed bowel dilation with no distal air. The clinical findings and presentation raised concern of bowel obstruction. On follow-up X-rays, a persistent radio opaque shadow in the lower abdomen raised the concern of urinary bladder obstruction. A Foley catheter was placed, obtaining 60 ml of urine. On day #4, an abdominal ultrasound was performed and revealed bilateral hydronephrosis, more prominent on the right side due to compression and a prominent parenchymal mass. A barium enema revealed a presacral soft tissue mass. On day #5 of life, an abdominopelvic computed tomography (CT) scan revealed a well-defined mass in the presacral space compressing the rectum and causing severe small and large bowel dilation. The mass was also causing compression of distal right ureter with severe hydroureteronephrosis and mild left hydroureter (see Figure 1). The CT scan findings favored a sacrococcygeal teratoma without evidence of metastasis. On day #6 of life, a pelvic magnetic resonance imaging (MRI) revealed a large hypervascular presacral mass measuring 3.2 cm anterior-posterior by 4.1 cm transverse by 6.4 cm (see Figure 2). The sacrum was preserved without evidence of erosion or bone invasion; however, the mass extended and expanded the left sacral vertebras 3 and 4 neural foramina. The MRI revealed bilateral hydronephrosis and bowel dilation, secondary to ureteral obstruction by the mass.

The patient was taken to the operating room on day #7 of life. Through a combined intra-abdominal pfannenstiel and sacral midline approach, the intrapelvic tumor was removed completely along with the coccyx. Sacral segments four and five needed removal due to adherence of the tumor. Dissection was undertaken staying near the capsule of the tumor avoiding damage to the already stretched pelvic nerves. During dissection, tumor adherence to the sacral promontory at the level of S1 to S3 caused piecemeal removal. Postoperatively, the child could move both lower extremities well, had pinprick, sphincter muscle stimulation in the anus, and did not develop urinary retention.

The tumor was received fixed in formalin and consisted of several fragments of gray tan soft tissue measuring in aggregate 8.1 × 6.5 × 1.1 cm. On section, the tumor was gray, tan, and soft with focal hemorrhages. A 1 cm segment of coccygeal bone was identified and was free of tumor. A microscopic examination disclosed a poorly differentiated neuroblastoma, with low mitotic karyorrhectic index. Few scattered ganglion cells were identified. The coccyx bone revealed no tumor involvement. Synaptophysin and chromogranin confirmed neuroblastoma. N-myc was not amplified.

## 3. Discussion

Congenital neuroblastoma is defined as a neuroblastoma identified within a month of birth [6]. Neuroblastoma is the most common extracranial solid tumor in infancy and childhood and can arise from any neural crest element of the sympathetic nervous system [7]. Neuroblastoma is slightly more common in boys than in girls, with a male to female ratio of 1.2 : 1 [8]. Some reports state that neonatal neuroblastoma constitutes about 1% of all neuroblastomas at all ages [9]. One study reported that 16% of infant neuroblastomas were diagnosed during the first month of life and 41% during the first 3 months [10]. In a study by Park et al., a prenatal diagnosis was usually made after 32 weeks of gestation and approximately 93% of tumors were adrenal in origin [5]. Some environmental factors have been implicated in the development of neuroblastoma (e.g., paternal exposure to electromagnetic fields or prenatal exposure to alcohol, pesticides, or phenobarbital) [5].

Prenatal ultrasound and postnatal ultrasound are a useful screening modality in the evaluation of congenital neuroblastoma [6], but sometimes it can be missed, as in this case where prenatal sonograms did not identify the mass. Neuroblastoma diagnosis is defined by pathologic confirmation from tumor tissue or by pathologic confirmation of neuroblastoma tumor cells in a bone marrow sample in the setting of increased urine or serum catecholamines or catecholamine metabolites [5].

In infants less than one year of age, about 55% of the tumors are intra-abdominal and about 30% are in the chest as compared to 75% and only 15%, respectively, in older patients [7]. In this report, the newborn patient presented at day #2 of life with findings suggestive of sepsis and abdominal distention secondary to an intra-abdominal mass causing compression of both the urinary and intestinal tracts. Around 20% of neonatal neuroblastoma presents with spinal cord compression requiring prompt diagnosis and treatment with steroids and chemotherapy to relieve the cord compression [11]. This patient did not present with spinal cord compression. Although sacrococcygeal neuroblastomas have low mortality. they have high morbidity owing to tumor bulk pressure and probable postoperative neurologic deficit [12].

The initial diagnostic testing should include CT or MRI to evaluate primary tumor size and regional extent and to assess for distant spread to neck, thorax, abdomen, or pelvic sites. Brain imaging is recommended only if clinically indicated by examination or neurologic symptoms. Bilateral posterior-iliac crest marrow aspirates and core biopsies are required to exclude marrow involvement [13]. Tumor markers or prognostic factors should be obtained prior to surgery but, in this case, were not obtained because it was thought to be a sacrococcygeal teratoma.

Few cases of sacrococcygeal neuroblastomas are reported in literature. Sunaa et al. [14] mentioned the first case of neonatal neuroblastoma mimicking Altman type III sacrococcygeal teratoma in 2005 [15]. It is worth noting that our case was thought to be a variant of teratoma. This is the first case reported of congenital intrapelvic neuroblastoma in Puerto Rico, in the early neonatal period. This case confirms the importance of the comprehensive physical examination and observation of low-risk newborn infants with history of an adequate prenatal care and unremarkable fetal ultrasonogram at midpregnancy. The differential diagnoses of abdominal distention in a newborn infant are broad, but prompt evaluation is important for the diagnosis and management of rare conditions presenting in the newborn period and the importance of pathologic confirmation of the diagnosis of tumor lesions in the newborn. A comprehensive and interdisciplinary approach when evaluating neonates with abdominal masses is also important for a prompt diagnosis and management of the case.
